# Cigarette smoke exposure decreases *CFLAR* expression in the bronchial epithelium, augmenting susceptibility for lung epithelial cell death and DAMP release

**DOI:** 10.1038/s41598-018-30602-7

**Published:** 2018-08-20

**Authors:** Alen Faiz, Irene H. Heijink, Cornelis J. Vermeulen, Victor Guryev, Maarten van den Berge, Martijn C. Nawijn, Simon D. Pouwels

**Affiliations:** 1 Department of Pathology and Medical Biology, University Medical Center Groningen, Groningen, The Netherlands; 2 GRIAC research institute, University of Groningen, Groningen, The Netherlands; 3 Department of Pulmonary Diseases, University Medical Center Groningen, Groningen, The Netherlands; 4 European Research Institute for the Biology of Ageing, Groningen, The Netherlands

## Abstract

Cigarette smoking is a major risk factor for the inflammatory disease, chronic obstructive pulmonary disease (COPD). The mechanism by which cigarette smoke (CS) induces chronic lung inflammation is still largely unknown. We hypothesize that immunogenic airway epithelial cell death is involved in the initiation of the inflammatory response. We previously identified *CFLAR*, the gene encoding the cell death regulator protein c-FLIP, to be associated with CS-induced release of damage-associated molecular patterns (DAMPs). Here, we investigated the effect of CS on expression levels of *CFLAR* in bronchial biopsies from smokers and non-smokers and *CFLAR* transcript isoform-expression in a dataset of air-liquid interface-differentiated bronchial epithelial cells. Furthermore, *CFLAR* was down-regulated by siRNA in lung epithelial A549 cells, followed by investigation of the effects on apoptosis, necrosis and DAMP release. CS exposure significantly decreased *CFLAR* expression in bronchial epithelial cells. Moreover, we observed a shift in relative abundance of the isoforms c-FLIP_S_ and c-FLIP_L_ transcripts in bronchial biopsies of current smokers compared to non-smokers, consistent with a shift towards necroptosis. *In vitro*, down-regulation of *CFLAR* increased apoptosis at baseline as well as CS extract-induced necrosis and DAMP release. In conclusion, CS exposure decreases *CFLAR* expression, which might increase susceptibility to immunogenic cell death.

## Introduction

Cigarette smoking is a major cause of morbidity and mortality worldwide, leading to various diseases including cardio-vascular diseases, lung cancer and chronic obstructive pulmonary disease (COPD)^1^. COPD is a chronic and progressive inflammatory pulmonary disease characterized by chronic bronchitis and emphysema. Only 20% of the smoking population develops COPD, indicating a role for genetic factors in the susceptibility for COPD, which is also supported by twin studies^2,3^. The mechanism by which inhalation of cigarette smoke (CS) induces chronic lung inflammation is still largely unknown. Recently, evidence is accumulating that dysregulated cell death processes and the subsequent release of Damage Associated Molecular Patterns (DAMPs) are involved in CS-induced neutrophilic airway inflammation and possibly in COPD^4–6^. Previously, we have shown that lung epithelial cells undergo immunogenic cell death, i.e. necrosis and necroptosis, rather than non-immunogenic, apoptotic cell death upon CS exposure, which coincides with the release of DAMPs and the subsequently induced innate immune response^5^. Chronic inhalation of CS induces cellular damage to airway epithelial cells, the first line of defense against inhaled pathogens and toxicants^7^, which may induce the release of DAMPs in genetically predisposed individuals^8^. Previously, we studied the effect of genetic susceptibility on CS-induced DAMP release in an *in vivo* murine model by exposing 28 inbred mouse strains to CS for five days and investigating the genetic regions associated with the release of DAMPs in bronchoalveolar lavage fluid^9^. Here, amongst others we identified a region associated with the gene *CFLAR*^9^, which encodes the apoptosis and necroptosis regulator protein c-FLIP. The *CFLAR* gene in humans is located on chromosome 2 in close proximity to several genes, including Caspase-8 and Caspase-10. The 14 exon *CFLAR* gene produces 13 splice variants of which three are expressed as proteins^10^, one 55 kDa long isoform (c-FLIP_L_) and two short isoforms, c-FLIP_S_, and c-FLIP_R_ (26 kDa and 24 kDa respectively). Whether c-FLIP_S_ or c-FLIP_R_ is produced is determined by the presence of the SNP rs10190751 (A/G)^11^. However, functionally both short isoforms are comparable. The c-FLIP proteins have a key role in the regulation of cell death. The c-FLIP protein is structurally closely related to the apoptosis regulator protein procaspase-8, although it lacks the catalytic function of caspase-8. All c-FLIP isoforms form heterodimers with caspase-8, limiting the functionality of caspase-8, reducing the execution of apoptosis^11,12^. However, while the short isoforms of c-FLIP are solely inhibitors of apoptosis, c-FLIP_L_ can both inhibit apoptosis when present in high concentrations and also promote apoptosis indirectly by increasing the enzymatic activity of caspase-8 when present in low concentration^10,13,14^. Next to a role in apoptosis c-FLIP is also involved in the modulation of regulated necrosis, termed necroptosis. Necroptosis is dependent of the formation of the ripoptosome protein complex. Upon activation of the ripoptosome protein RIPK1, the necroptosis-inducing necrosome is formed and RIPK3 is activated. RIPK3 phosphorylates Mixed Lineage Kinase domain-Like protein (MLKL) leading to pore formation in the plasma membrane. Here, c-FLIP isoforms have dual functions, as c-FLIP_S_ promotes the assembly of the ripoptosome and therefore necroptosis, while c-FLIP_L_ inhibits this process^14^.

It has been shown that necroptosis is dysregulated in COPD^5,15^, although the mechanisms and consequences for pulmonary inflammation are still largely unclear. *CFLAR* has been identified as a susceptibility gene for CS-induced neutrophilic airway inflammation^9^, but no studies have been performed investigating the role of *CFLAR* in COPD and CS-induced pulmonary inflammation. We hypothesize that *CFLAR* expression is decreased upon smoking which increases the susceptibility for CS-induced epithelial immunogenic cell death with subsequent DAMP release, which may induce pulmonary inflammation in COPD patients. Here, we investigated the effect of CS exposure on differentiated primary bronchial epithelial cells *in vitro* using publically available datasets. Further, we studied the functional effect of *CFLAR* down-regulation on CS-induced cell death and DAMP release in an alveolar epithelial cell line *in vitro*. Lastly, we investigated bronchial *CFLAR* expression and *CFLAR* splice variant abundance in bronchial biopsies from current smokers and non-smokers.

## Results

### CFLAR expression is downregulated upon cigarette smoke exposure

**Figure 1:**
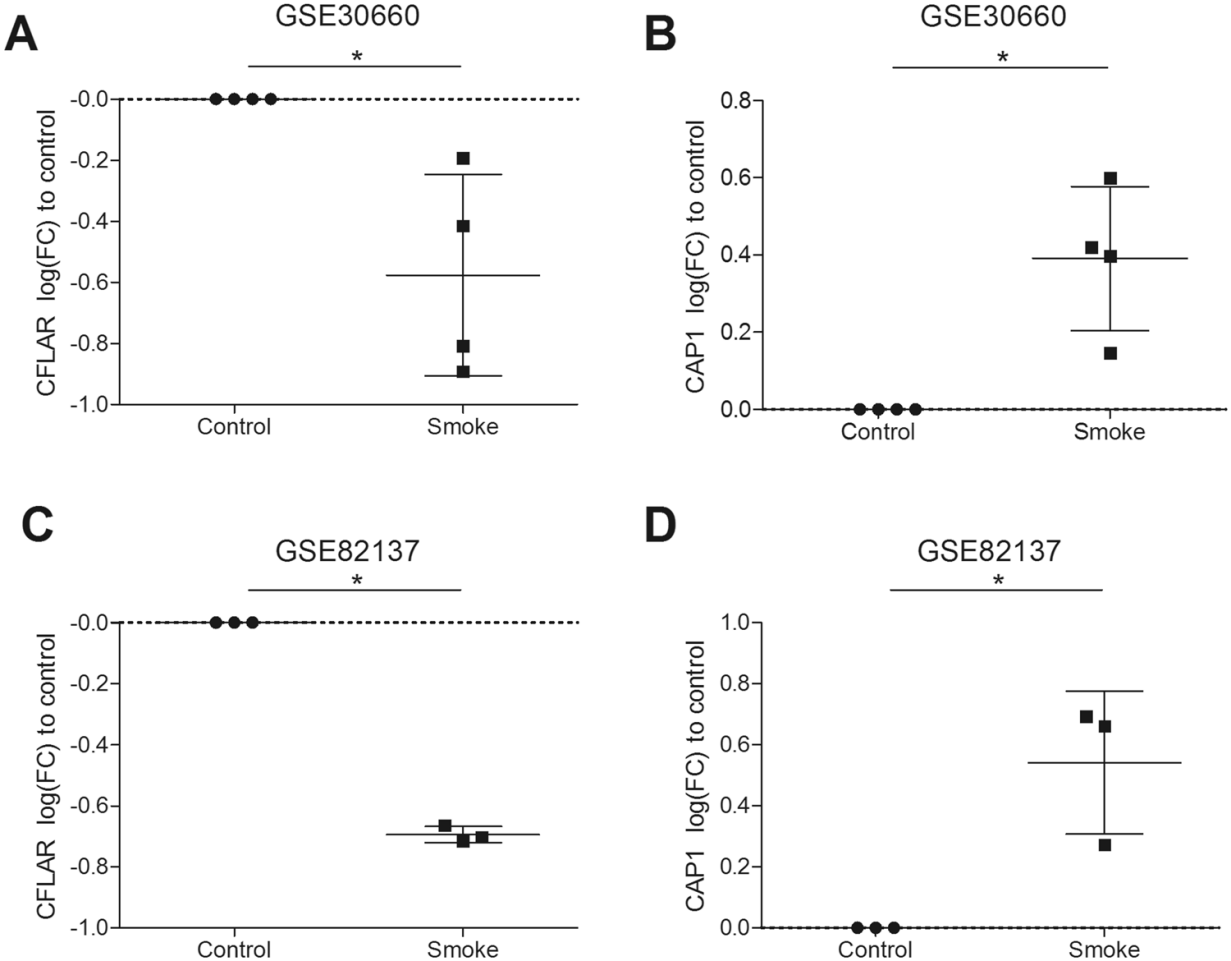
CFLAR and CAP1 expression is altered upon smoke exposure. CFLAR expression measured by microarray in bronchial epithelial cells grown at air liquid interface (ALI) and exposed to gaseous cigarette smoke (**A**,**B**) 30 minutes exposure to whole cigarette smoke or no challenge (n = 4) on four separate days and (**C**,**D**) 48 minutes of exposure on one day with whole cigarette smoke or air and then rested for 24 hours (n = 3). Gene expression is shown as log2 (fold change) compared to control. All data is shown as means ± SEM. Significance was tested using a Mann-Whitney U test, *P < 0.05.

**Figure 2:**
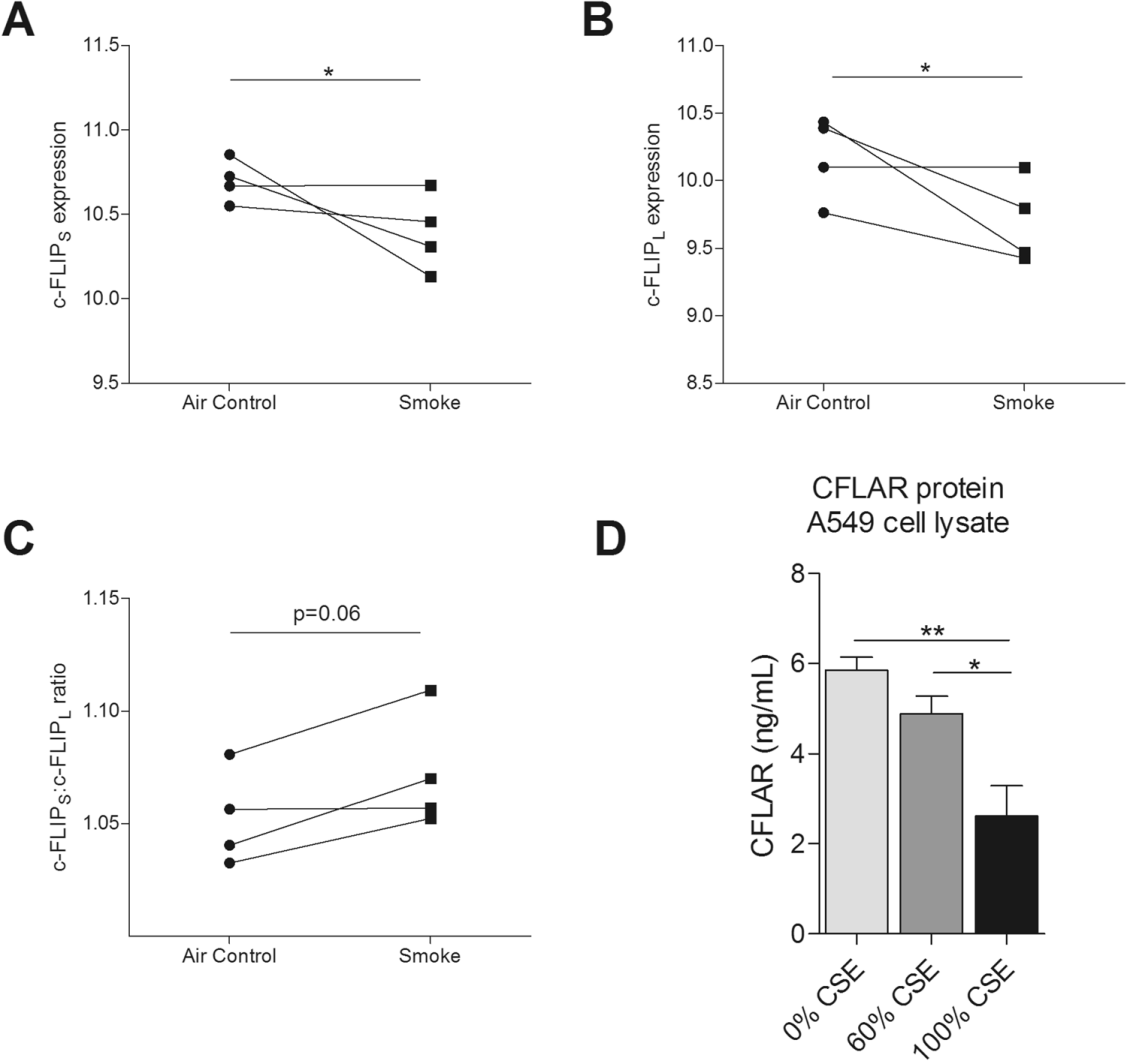
Expression and ratio of c-FLIP_S_ and c-FLIP_L_ during smoke exposure in airway epithelial cells grown at air liquid interface. (**A**) c-FLIP_S_ and (**B**) c-FLIP_L_ expression measured by microarray in bronchial epithelial cells grown at air liquid interface (ALI) and exposed to gaseous phase cigarette smoke for 30 minutes or no challenge (n = 4) on four separate days. (**C**) Ratio of c-FLIP_S_:c-FLIP_L_ in the presence and absence of smoke exposure. (**D**) A549 cells were exposed to 0, 60 or 100% cigarette smoke extract (CSE) for 4 hours and incubated with fresh, CSE-free medium for 24 hours afterwards. Intracellular CFLAR protein levels were measured in cell lysate using a commercially available ELISA kit. All data is shown as means ± SEM. Significance was tested using a Mann-Whitney U test, *P < 0.05, **P < 0.01.

Initially, to investigate whether the expression of genes previously identified in our mouse GWAS analysis^9^ are modified by CS exposure, we accessed their expression in two independent, publically available datasets of bronchial epithelial cells from healthy individuals differentiated at air liquid interface (ALI). Cells were exposed to gaseous CS or air, either for 30 minutes (n = 4) on four separate days (NCBI accession number: GSE30660) or 48 minutes on one day (n = 3) and then rested for 24 hours (NCBI accession number: GSE82137). We analyzed these datasets and observed that the expression of *CFLAR* and Adenylyl cyclase-associated protein 1 (*CAP1*) was significantly decreased and increased, respectively upon gaseous CS exposure compared to air exposure in both datasets (p < 0.05, Fig. 1A–D, Table 1). Both c-FLIP_S_ and c-FLIP_L_ were significantly decreased by smoke exposure (Fig. 2A,B). Furthermore, we found a trend for an increase of the c-FLIP_S_:c-FLIP_L_ ratio reflecting what was observed in the bronchial biopsies (Fig. 2C). Considering the known role of *CFLAR* in cell death we focused on *CFLAR* in the functional and validation experiments. To confirm the CS-induced decrease in CFLAR expression on protein level we exposed human alveolar A549 cells to several concentrations of CSE. Here. we observed a dose-dependent decrease in the CFLAR protein expression (Fig. 2D).

**Table 1:** Genes altered by cigarette smoke exposure.

	GSE82137		GSE30660	
	logFC	pvalue	logFC	pvalue
AOX2P	NA	NA	0.043672	0.742
**CFLAR**	**−0.69487**	**2.41E-05**	**−0.5772**	**0.0022**
ARHGEF11	−0.15231	0.136	0.229575	0.0312
PPT1	−0.36431	0.00267	0.099934	0.243
**CAP1**	**0.541592**	**0.000443**	**0.390945**	**0.00384**
DSCAML1	−0.20422	0.0831	−0.44004	0.00135
ELAC2	−0.01729	0.884	−0.0406	0.699
ARHGAP44	−0.14841	0.236	−0.36535	0.0778
MYOCD	0.104138	0.32	0.148621	0.265
TRIP11	−0.23022	0.139	0.14179	0.0844
ENOX1	−0.0976	0.363	−1.21397	0.000015

Bold indicated genes significant in the same direction in both datasets.

### CFLAR downregulation induces apoptosis at baseline and increases CS-induced necrosis in A549 cells

**Figure 3:**
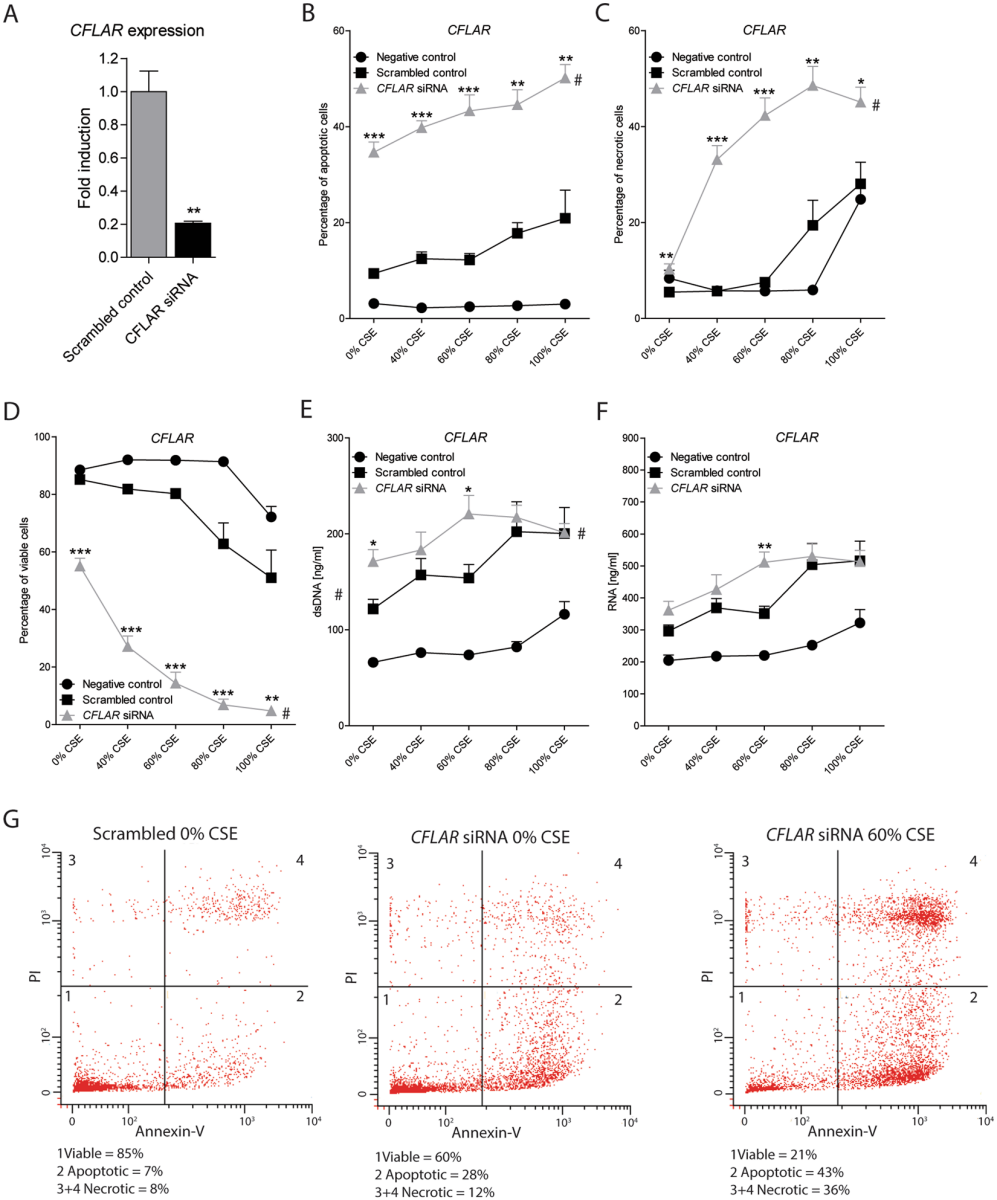
CFLAR downregulation induces apoptosis and increases the susceptibility for cigarette smoke-induced necrosis and DAMP release in A549 cells. Cells were seeded in duplicates, grown to 60% confluence, transfected with CFLAR targeting siRNA oligo’s or scrambled control, grown for another 48 hours, serum deprived overnight and exposed to CSE for 4 hours. Subsequently CSE was washed away and replaced by CSE and serum free medium for 24 hours. (**A**) The mRNA expression of CFLAR compared with scrambled control is shown in A549 cells upon treatment with specific siRNA for three days, as analyzed by quantitative RT-PCR. Data is shown as fold induction of mRNA expression of A549 cells treated with scrambled siRNA (2^−ΔΔCt^) compared to A549 cells treated with siRNA specific for CFLAR (2^−ΔΔCt^). Negative control represents non-transfected cells. The levels of (**B**) apoptotic, (**C**) necrotic and (**D**) viable cells were analyzed in A549 cells upon exposure to 0–100% CSE using Annexin-V/PI staining for flow cytometry. The levels of (**E**) dsDNA and (**F**) RNA were measured in cell free supernatant of A549 cells exposed to 0–100% CSE. (**G**) Graphical representation of FACS plots. All data is shown as mean ± SEM. Significance was tested between scrambled control and CFLAR siRNA treated cells using a Mann-Whitney U test, *p < 0.05, **p ≤ 0.01, ***p ≤ 0.001. Significance between the scrambled control and the siRNA-treated cells over the range of concentrations was tested using a two-way ANOVA with Bonferroni correction, ^#^p < 0.05.

In order to study the functional effects of *CFLAR* downregulation, specific *CFLAR* siRNA assays were used to decrease the *CFLAR* mRNA expression in the alveolar epithelial cell line A549, resulting in a reduction of *CFLAR* expression by approximately 40% (Fig. 3A). Next, A549 cells were exposed to a range of 0–100% cigarette smoke extract (CSE) to study the effect of *CFLAR* downregulation on CSE-induced cell death and DAMP release. *CFLAR* downregulation strongly increases the levels of apoptotic cells from 9.39% (±0.41) to 34.64% (±2.15) at baseline (Fig. 3B). Upon CSE exposure, the level of apoptotic cells shows a further increase in both the *CFLAR* siRNA and the scrambled control treated cells, indicating that *CFLAR* downregulation does not affect the sensitivity to CSE with respect to the induction of apoptosis. Furthermore, the percentage of CSE-induced necrotic cells was significantly increased upon *CFLAR* downregulation (Fig. 3C). The percentage of necrotic cells increased upon exposure to ≥60% CSE in the scrambled control, while in the *CFLAR* downregulated cells, a stronger and more rapid increase in the percentage of necrotic cells was observed. This, indicates that *CFLAR* downregulation increases the susceptibility for CS-induced necrosis. Pretreatment with the necroptosis inhibitor Necrostatin-1 did not alter the amount of CSE-induced necrotic cells upon *CFLAR* downregulation *(data not shown)*, suggesting that the majority of cells dies in a necrotic rather than a necroptotic fashion upon exposure to cigarette smoke. Lastly, upon increasing concentrations of CSE, the levels of extracellular dsDNA and RNA increased in a dose-dependent manner (Fig. 3E,F). *CFLAR* downregulation further increases the levels of extracellular DAMP release, especially upon 60% CSE exposure. Together, *CFLAR* downregulation increases the baseline level of apoptosis as well as the susceptibility for CSE-induced necrosis and subsequent DAMP release in A549 cells.

### CFLAR expression and splicing is altered during *in-vivo* smoke exposure

**Figure 4:**
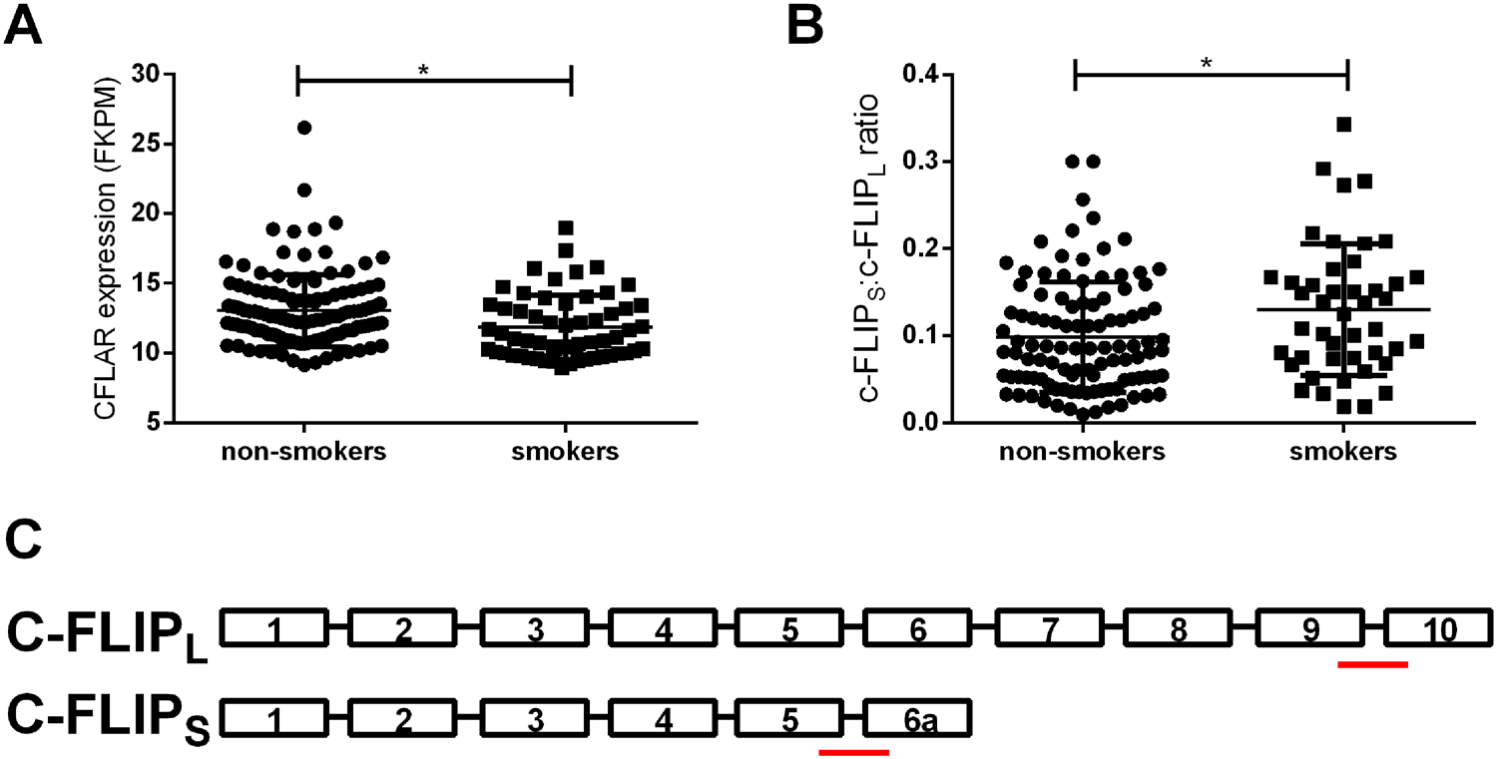
CFLAR expression and splicing is altered by smoking. (**A**) CFLAR expression Fragments Per Kilobase Million (FKPM) in bronchial biopsies from non- (n = 125) and current smokers (n = 59). Ratio of c-FLIP_S_ and c-FLIP_L_ in bronchial biopsies of non- (n = 107) and current-smokers (n = 47). (**C**) Graphical representation of c-FLIP_S_ and c-FLIP_L_ exon structure. Underlined regions indicate unique regions measured by RNA-Seq to determine the expression level of c-FLIP_S_ and c-FLIP_L_ individually. Individuals with an absence of either c-FLIP_L_ and c-FLIP_S_ expression were excluded from this analysis.

We next investigated whether *CFLAR* and *CAP1* expression was altered in bronchial biopsies of current-smokers (n = 59) and non-smokers (n = 125) not taking inhaled corticosteroids (ICS). An outline of the patients’ characteristics is shown in Table 2. We found a significant increase in *CAP1* gene expression (logFC = 0.21, p = 0.002), while a decrease for *CFLAR* expression was shown in current smokers compared to non-smokers, which is in line with the effect of CS exposure in bronchial epithelial cells *in vitro* (p < 0.05, Fig. 4A). In order to evaluate the ratio of c-FLIP_S_ and c-FLIP_L_ we analyzed the expression of specific splice sites unique to each transcript (c-FLIP_S_ spanning exon5-exon6a; c-FLIP_L_ spanning exon9-exon10, Fig. 4C). Interestingly, we found that the ratio of c-FLIP_S_: c-FLIP_L_ was higher in current smokers compared to non-smokers (p < 0.05, Fig. 4B). These results indicate that there is an overall decrease in *CFLAR* expression in smokers, indicating less suppression of apoptosis, while the increased ratio of c-FLIP_S_: c-FLIP_L_ suggesting a shift towards necroptosis.

**Table 2:** Patient characteristics.

Characteristics	Non-smoker	Smoker
N	125	59
PC20AMP (mg/mL) median [min, max]	866.6 [0, 10314]	476.9 [0.02, 9072]*
FEV_1_ (%pred) mean [sem]	93 [1.44]	93.1 [1.98]
ICS dose (μg/day) median [min, max]	800 [28, 2000]	800 [200, 1000]
Beta-agonist use (n(%))^#^	42 (34)	11 (19)
Sex (m/f)	58/67	37/22
Age (years) mean [sem]	45.2 [1.35]	43 [1.93]
FEV_1_/FVC (%) mean [sem]	75 [0.85]	75.2 [1.06]
Reversibility FEV_1_ (%) mean [sem]	6.3 [0.51]	5.7 [0.66]
Atopy (skin prick) (n(%))^#^	21 (46)	26 (30)
Atopy (phadiatop) (n(%))^#^	25 (70)	4 (80)
Blood eosinophils (10^9^/L) median [min, max]	0.15 [0, 0.9]	0.18 [0, 0.49]
Asthma status (asthma/healthy)^#^	85/40	22/37*
Packyears median [min, max]	0	15.8 [0, 52.0]*

N = number of participants, PC_20_AMP = Concentration of Adenosine 5-monophosphate causing the FEV_1_ to drop 20%, ICS = inhaled corticosteroids, sem = standard error of the mean. Student T-test was conducted unless stated otherwise, * = p < 0.05. # = p < 0.05 using the Chi square test.

## Discussion

In the current study, we showed for the first time that the expression of *CFLAR* in the airways is decreased upon CS exposure both *in vitro* and *in vivo*. Moreover, downregulation of total *CFLAR* in pulmonary epithelial cells increases apoptosis at baseline and necrosis and DAMP release upon CSE exposure. Lastly, we show that the ratio of c-FLIP_S_: c-FLIP_L_ is increased in bronchial biopsies from current smokers compared to non-smokers, suggesting a shift towards necroptosis.

Next to *CFLAR* also the mRNA expression of *CAP1* was found to be significantly changed in two independent studies upon CSE exposure. *CAP1* encodes Cyclase Associated Actin Cytoskeleton Regulatory Protein 1, a protein that is involved in actin filament dynamics and cellular migration^16^. Little is known about the role of *CAP1* in cigarette smoking or COPD, although one study showed that *CAP1* expression is decreased in lung tissue of COPD patients^17^. In contrast, we show that exposure of differentiated primary bronchial epithelial cells to CSE increases the expression of *CAP1*, suggesting a protective mechanism of increased *CAP1* expression which is dysregulated in COPD patients, which will be of interest for future studies.

The human *CFLAR* gene encodes the apoptosis regulator protein c-FLIP, which can be found as three major protein forms, produced by alternative splicing, the long variant c-FLIP_L_, and two short variants c-FLIP_s_ and c-FLIP_R_^10^. Together these isoforms hold a key position in the regulation of cell death. One way c-FLIP does this is by forming heterodimers with Caspase-8^11^. In the current study we found that the downregulation of total *CFLAR* increases apoptosis at baseline. This is in agreement with previous *in vitro* studies which have found that all isoforms of c-FLIP are inhibitors of apoptosis, however c-FLIP_L_ can also promote apoptosis depending on the concentration^11,13^. Next to apoptosis c-FLIP is also involved in the regulation of necroptosis. Our treatment of A549 cells with increasing levels of CSE leads to increased levels of necrosis, however we were unable to differentiate whether this was planned necroptosis or necrosis. Previous findings from our group have shown that CSE induces a switch from apoptosis to necrosis in bronchial epithelial cells^18^. Furthermore, in follow-up studies we observed that this process of CSE-induced bronchial epithelial cell death is in fact necroptosis^5^. Also here the complexity of c-FLIP is present as c-FLIP_L_ inhibits this process, while c-FLIP_S_ promotes necroptosis^10^. Previously, we showed that out of a panel of six different DAMPs, dsDNA has the strongest correlation with cigarette smoke-induced airway inflammation in mice^19^. Moreover, this DAMP is specifically released upon cell death and a reliable marker for CSE-induced DAMP release from primary bronchial epithelial cells^8^. In the current study we observed an increase in dsDNA and RNA release in *CFLAR* deficient cells upon CSE exposure. The fact that the observed increase in CS-induced DAMP release in *CFLAR* down-regulated cells is statistically significant yet less striking than could have been expected is likely to due to an increase in DAMP release induced by the transfection itself (scrambled control), which may reduce the potency to further enhance CS-induced DAMP release. Furthermore, our transfection efficiency was limited and induced approximately 40% *CFLAR* downregulation, and although we were still able to modulate CSE-induced DAMP release and cell death, higher transfection efficiency possibly increases the effects on CSE-induced DAMP release. It has been well established that upon CS-induced necroptosis and/or necrosis DAMPs are released, which may initiate or potentiate an inflammatory response in the airways of COPD patients upon cigarette smoking^4,8^. Interestingly, we found that *CFLAR* is not only decreased by CS exposure of airway epithelial cells *in vitro*, but is also decreased *in vivo* as evident from the airway wall biopsies from current-smokers compared to non-smokers.

However, despite this overall decrease in *CFLAR* we also observed a shift in the balance between c-FLIP_S_ and c-FLIP_L_ during smoking. This shifted balance between c-FLIP_S_: c-FLIP_L_ has previously been associated with the regulation of both apoptosis and necroptosis, with a decrease shifting the cells fate towards apoptosis, while an increase directs the cells fate necrosis^20^. The increased ratio of c-FLIP_S_: c-FLIP_L_ observed in bronchial biopsies from current smokers compared to non-smokers identified in the current study, may indicate a tendency of airway wall structural cells towards immunogenic necroptosis, leading to or contributing to the well documented inflammation associated with smoking and COPD^21^. However, the response of the lung epithelium towards CS is unlikely to be solely dependent on CFLAR, and further studies need to address whether and which other genes contribute to the dysregulated epithelial cell death response upon smoking in COPD. Furthermore, one of the limitations of the current study is that we cannot exclude that asthma status may have an influence on gene expression of *CFLAR* and *CAP1*.

In conclusion, our study shows for the first time that exposure of airway epithelial cells to CS decreases the expression of *CFLAR* both *in vitro* and *in vivo*. While the overall ratio of c-FLIP_S_: c-FLIP_L_ is increased upon smoking. This decrease in *CFLAR* and shift in c-FLIP_S_: c-FLIP_L_ ratio may increase the susceptibility of cells to underdo necroptosis upon CS exposure and subsequently release DAMPs. These DAMPs may then potentiate the inflammatory response in the airways. Dysregulation of c-FLIP and the cell death machinery may be involved in CS-induced airway inflammation and related pathologies.

## Materials and Methods

### Bronchial biopsies processing for quantification of CFLAR expression

Bronchial biopsies were collected from respiratory healthy subjects^22^ and current asthma patients^23,24^ with a previous doctor’s diagnosis of asthma, documented reversibility and airway hyper responsiveness to histamine (PC20 ≤ 32 mg/mL). All study protocols were approved by the UMCG medical ethics committee and all subjects provided written informed consent. All clinical procedures conformed to the standards set by the latest Declaration of Helsinki. RNA was isolated and sequenced as described in the online supplement.

### RNA extraction, sample preparation and high-throughput sequencing

Bronchial biopsies were taken from segmental divisions of the main bronchi. Biopsies frozen in Tissue-Tek (VWR, Radnor, PA) at −80 °C were thawed at room temperature and cut from the blocks when they were semi-solid. Total RNA was extracted using AllPrep DNA/RNA Mini kit (Qiagen, Venlo, the Netherlands). Samples were lysed in 600 µl RLT-plus buffer using an IKA Ultra Turrax T10 Homogenizer, and RNA was purified according to the manufacturer’s instructions. RNA samples were dissolved in 30 µl RNAse free water. Concentrations and quality of RNA were checked using a Nanodrop-1000 and run on a Labchip GX (PerkinElmer, Waltham, MA).

RNA samples were further processed using the TruSeq Stranded Total RNA Sample Preparation Kit (Illumina, San Diego, CA), using an automated procedure in a Caliper Sciclone NGS Workstation (PerkinElmer, Waltham, MA). In this procedure, all cytoplasmic and mitochondria rRNA was removed (RiboZero Gold kit). The obtained cDNA fragment libraries were loaded in pools of multiple samples unto an Illumina HiSeq2500 sequencer using default parameters for paired-end sequencing (2 × 100 bp).

### Gene expression quantification

The trimmed fastQ files where aligned to build b37 of the human reference genome using HISAT (version 0.1.5) allowing for 2 mismatches^24^. Before gene quantification SAMtools (version 1.2) was used to sort the aligned reads^25^. The gene level quantification was performed by HTSeq (version 0.6.1p1) using Ensembl version 75 as gene annotation database.

### Quality Control

Quality control (QC) metrics were calculated for the raw sequencing data, using the FastQC tool (version 0.11.3). Alignments of 220 subjects were obtained. QC metrics were calculated for the aligned reads using Picard-tools (version 1.130) (http://picard.sourceforge.net) CollectRnaSeqMetrics, MarkDuplicates, CollectInsertSize-Metrics and SAMtools flagstat. We discarded 36 samples due to poor alignment metrics. In addition, we checked for concordance between sex-linked (*XIST* and Y-chromosomal genes) gene expression and reported sex. All samples were concordant. This resulted in high quality RNAseq data from 184 subjects.

### Differential expression

Raw counts of expressed features were analyzed using the R-package DESeq2^25^. Feature counts were set as the dependent variable, smoking status was investigated correcting for age and gender. The use of splice sites was quantified by counting split reads mapping across exon-exon junctions using a custom in-house script (available upon request).

### CFLAR gene expression analysis

Two publically available microarray data sets from airway epithelial cells grown at air liquid interface (ALI) from healthy controls and treated with gaseous whole smoke were analyzed (GSE30660, n = 3; and GSE82137, n = 4). ALIs from the GSE30660 dataset were exposed for 30 minutes on four separate days with whole cigarette smoke (n = 4) compared to air exposure, while ALIs from the GSE82137 dataset were treated with a 48 minutes exposure on day one with whole cigarette smoke and then rested for 24 hours, compared to air exposure. Microarray analysis was conducted using R software version 3.02, using the Bioconductor-limma package, and normalized using Robust Multi-array Average (RMA). A paired linear analysis was conducted using limma comparing treatment vs control.

The influence of smoking was investigated in probes specific for c-FLIPS and c-FLIP_L_ were investigated in the GSE82137 dataset. Furthermore the ratio between these probes was investigated in the presence and absences of smoke exposure.

### Cell culture and CSE stimulation

The human adenocarcinoma alveolar cell line A549 was cultured in RPMI-1640 supplemented with 10% fetal calf serum (FCS; Biowhittaker, Verviers, Belgium), 100 U/ml penicillin and 100 mg/ml streptomycin. Cells were grown to confluence and serum-deprived overnight before use. Cigarette smoke extract (CSE) was prepared as described before with two filterless Kentucky 3R4F research-reference cigarettes and a Watson Marlow 603S smoking pump at a rate of 8 L/hr (Watson-Marlow, Delden, The Netherlands)^5,8^. The 100% CSE mixture was prepared by bubbling the CS of two cigarettes through 25 mL of RPMI-1640 medium supplemented with 100 U/ml penicillin and 100 mg/mL streptomycin. This solution was diluted in growth medium to the desired concentration.

### siRNA transfection

*CFLAR* down-regulation was performed using commercially available siRNA assays according to manufacturer’s protocol (CFLAR MISSION® esiRNA, Sigma-Aldrich, Saint-Louis MO, USA), using RNAiMAX lipofectamine as a transfection reagent (Invitrogen, Carlsbad CA, USA). Cells were seeded in duplicates, grown to approximately 60% confluence, transfected with siRNA or scrambled control, grown for another 48 hours, serum deprived overnight and exposed to CSE for 4 hours. Subsequently CSE was washed away and replaced by CSE and serum free medium for 24 hours. The levels of the DAMPs dsDNA and RNA were measured in cell free supernatant using the Quant-iT™ Pico- and Ribo-Green® dsDNA Assay Kits respectively (Invitrogen). The percentage of viable, apoptotic and necrotic cells were determined using an Annexin-V (Immunotools, Friesoythe, Germany) and Propidum Iodide (PI; Sigma-Aldrich, Saint Louis, USA) staining for flow cytometry. Annexin-V/PI double negative cells were designated as viable cells, Annexin-V positive and PI negative cells were designated as apoptotic and all PI positive cells, either Annexin-V positive or negative, were designated as necrotic cells.
